# Recent Progress in Gain Materials for Microlasers and Modern Digital Approaches for Biophotonics: From Dyes to Semiconductors

**DOI:** 10.3390/mi17030366

**Published:** 2026-03-18

**Authors:** Carlos A. Calles-Arriaga, Romeo Selvas-Aguilar, Arturo A. Castillo-Guzmán, Wilian J. Pech-Rodríguez, Enrique Rocha-Rangel, María T. Maldonado-Sada, José A. Rodríguez-García, José A. Castillo-Robles, Eddie N. Armendáriz-Mireles

**Affiliations:** 1 Research and Postgraduate Department, Victoria Polytechnic University, Science and Technology Park, Victoria City 87138, Tamaulipas, Mexico; wpechr@upv.edu.mx (W.J.P.-R.); erochar@upv.edu.mx (E.R.-R.); jrodriguezg@upv.edu.mx (J.A.R.-G.); jcastillor@upv.edu.mx (J.A.C.-R.); earmendarizm@upv.edu.mx (E.N.A.-M.); 2 Facultad de Ciencias Físico Matemáticas, Universidad Autónoma de Nuevo León, Av. Universidad s/n, Ciudad Universitaria, San Nicolás de los Garza 66455, Nuevo León, Mexico; romeo.selvasag@uanl.edu.mx (R.S.-A.); arturo.castillogz@uanl.edu.mx (A.A.C.-G.); 3 School of Engineering and Science, Autonomous University of Tamaulipas, Victoria City 87149, Tamaulipas, Mexico; mmaldonado@uat.edu.mx

**Keywords:** lasers, dyes, semiconductors, machine learning, neural networks

## Abstract

Microlasers are innovative photonics devices that have recently attracted attention for their unique characteristics, including compactness, broad spectral emission, and low lasing threshold. These properties are beneficial in biophotonics as these lasers can interact with biological materials without causing damage, especially for optical biosensing applications. Among the optical materials recently used as gain media in microlasers are organic dyes, rare-earth ions, fluorescent proteins, and semiconductors, including quantum dots and perovskites. Moreover, different optical cavities and current laser configurations have increased the versatility of microlasers. Recently, digital sensing methods based on novel algorithms, machine learning, and neural networks have been combined with microlaser systems to enhance their accuracy and expand their applications. This work provides a comprehensive review of recent progress in microlasers, covering gain media, microcavity types, and their applications in biophotonics, including conventional spectral-based sensing and new digital approaches for the biomedical field.

## 1. Introduction

Laser is the acronym for light amplification by stimulated emission of radiation. Basically, a laser is an amplified beam of light with special characteristics, such as high directionality, monochromaticity, and coherence. High directionality allows a laser beam to propagate through a medium with very small divergence. This results in a small spot-size variation that is often in the micrometer range. Monochromatic light from a laser is an emission in a very narrow linewidth that is usually of the order of nanometers. In visible lasers, monochromaticity is observed as the emission of a very specific color. Coherence refers to the fixed distribution of light waves. In a laser, all the waves are in phase, resulting in coherent light with high brightness. These characteristics make the laser a unique tool for applications requiring high accuracy and resolution.

Laser technology has become omnipresent in multiple sectors such as optical communications [1], advanced manufacturing [2], biology [3], and medicine [4]. In the biomedical field, lasers are reliable tools for diverse applications, including surgery [5], fluorescence microscopy [6], photodynamic therapy [7], and flow cytometry [8]. However, most current technology relies on macro lasers, which are bulky and consume significant energy. In recent years, microlaser research has attracted attention mainly due to their small form factor and low lasing threshold, making them suitable for micro-scale biological sensing.

The general structure of a microlaser consists of the same elements as a conventional laser: gain medium, optical cavity, and pump source. Recently, several materials have been studied as gain media. Organic dyes have attracted interest because their synthesis process is simple. Light amplification in organic dyes is primarily implemented in optofluidics systems in pulsed mode [9]. Rare-earth ions from the lanthanide family are another attractive option, as their lasing properties have been widely studied for bulk and fiber lasers with wavelength emissions ranging from ultraviolet (UV) to infrared (IR) [10]. Semiconductor-based gain media, including perovskites and quantum dots, are also part of the current trends in this research area [11].

Optical cavities are critical in laser performance. Fabry–Perot (FP) cavities are typically used in commercial lasers. FP cavities consist of two reflecting surfaces that provide light amplification from a gain medium. In microlasers, some optical cavities are directly integrated into the gain media, for example, in whispering gallery mode (WGM) microspheres [12]. In other cases, a special optical fiber cavity, such as fiber Bragg gratings (FBGs), could be used as an FP microresonator [13].

**Figure 1:**
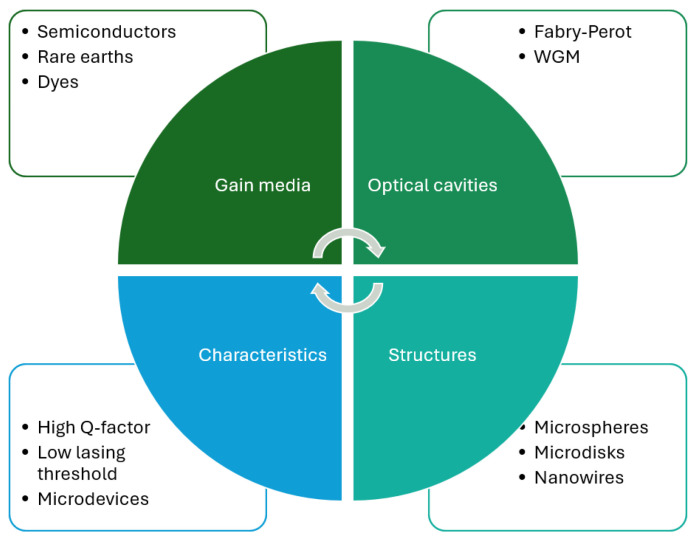
Examples of optical materials, resonators, characteristics, and microstructures used in microlasers [18,19,20].

In addition to their small size, microlasers also exhibit Q factors exceeding 10^3^ [14]. This parameter is fundamental because it directly affects the emission linewidth, which favors their implementation as spectral sensors [15,16]. Another key characteristic is their low lasing threshold below 140 kW/cm^2^ [17]. This feature is critical not only for efficiency but also for its applications in biophotonics, where optical power should be maintained at lower levels to avoid damage to biological organisms. Figure 1 shows an overview of microlaser characteristics.

Some insightful reviews of microlaser schemes have been published previously. For example, Feng and Bai [21] published an article emphasizing optofluidic microcavities for biosensors. However, this study was published in 2018. More recently, in 2021, Toropov et al. [22] published a comprehensive review describing the latest biosensing mechanisms based on WGM microlasers, including emerging materials. Since then, several new microlaser studies have been reported consisting in new materials, optical cavities, sensing techniques, and applications. This paper includes several optical materials for microlasers, such as halide perovskites, quantum dots, rare earths, optical fibers, and advanced materials, which have found diverse applications in biomedical fields. Other recent articles have focused on bioapplications [23,24], but they do not provide an analysis of optical fiber devices or modern approaches, such as machine-learning-based digital sensing.

This paper reviews the latest developments in microlaser systems applied to biophotonics. The second section presents an in-depth examination of the optical materials that have recently been used as gain media. This section discusses the physical and chemical properties of organic and inorganic optical materials. The third section analyzes typical optical microcavities, focusing on key aspects such as stability and beam quality. The fourth section discusses non-conventional configurations in microlasers. A comprehensive study of current applications of microlasers in biology and biomedical engineering is presented in the fifth section. Modern digital approaches based on machine learning are described in the sixth section. The following section provides an in-depth discussion of current trends, including the advantages and limitations of microlasers. Moreover, further work and perspectives are explored. In the last section, conclusions and final remarks are outlined.

## 2. Gain Media

The gain medium plays a key role in the performance of any laser. In microlasers, the gain medium can range from an organic dye solution to a solid, such as a semiconductor. In recent years, several studies on gain media, such as rare earths, biomaterials, perovskites, and quantum dots, have garnered attention due to their versatility in emission wavelengths and stability, making them suitable for biomedical applications. This section provides the main characteristics of gain media used for light amplification in microlasers.

### 2.1. Dyes

A dye laser is a type of laser with a gain medium based on a dye that is often in solution but also in solid or vapor form. In microfluidics, solutions often consist of solvents, such as alcohol, and low concentrations of the active dye. Dye lasers operate mainly in the visible region and exhibit a high tuning range, although they can also operate in the UV [25] and IR [26] zones. However, because the gain media may be degraded due to photobleaching, output powers are limited to several watts, though they can be used in pulsed mode to increase peak power. Some applications of this kind of laser include optical spectroscopy for physical–chemical analysis and medical procedures, the laser cooling of molecules, and photodynamic therapy [27,28]. Some gain media, for example, rhodamine or chlorophyll, previously used in conventional dye lasers, have recently been implemented in microlasers based on microcrystals, microfluidics, and optical fibers, among others. Table 1 lists different types of dyes used as gain media in microlasers.

**Figure 2:**
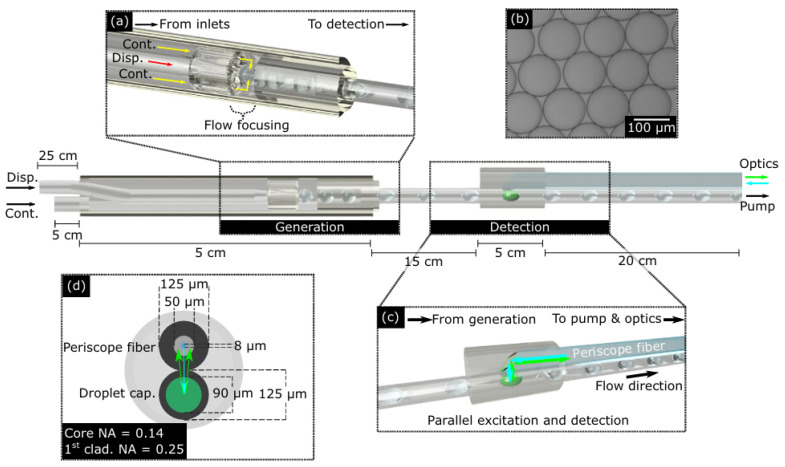
Fiber optofluidic laser (FOFL) based on induced fluorescence [39]. (**a**) Droplet generation in a five-hole capillary. (**b**) Droplets from fluorescein. (**c**) Fluorescence emission detection with a double-clad fiber as a periscope fiber. (**d**) Internal section view of the periscope fiber and the droplet capillary.

Typical dye-based optofluidics experimental setups are based on chips fabricated on substrates. However, in recent years, optical fibers have also been used for this purpose in optofluidic lasers (OFLs) [38]. Optical fiber can be made from silica or polymethylmethacrylate with a core-cladding structure. The fiber laser has several advantages over bulk lasers, such as better thermal distribution, immunity to electromagnetic interference, and higher efficiency, among others. The fiber optofluidic laser (FOFL) combines the attributes of the optical fiber at the microscale with the versatility of using a fluid as the gain medium with a wide emission spectrum. This combination broadens their potential application in biophotonics. Parker et al. [39] developed a compact fiber-optic device utilizing microfluidics to detect viruses. As shown in Figure 2, the detection module of about 333 µm consists of a modified double-clad fiber with fluorescein dye excited by a 488 nm fiber laser. Zhang et al. [33] studied an optofluidic laser to measure hemoglobin (Hb) concentration. The device is based on a hollow optical fiber (HOF) that is used as a microfluidic channel. In this case, rhodamine 6G (R6G) was used as the gain medium for the microlaser. The operating principle is based on fluorescence changes resulting from the interaction between the amplifier medium in the fiber and Hb. One important advantage of optical fiber sensors, besides their compactness in the micrometer range, is that they can easily be integrated into optical communication systems to transfer information from devices to station works or sensing networks [40].

Fluorescent dyes can be combined with cells to produce a cell laser. An experimental setup consists of a cell containing a fluorescent dye as the gain medium placed in an optical cavity [41]; pump energy is provided by a light source. One advantage of cell lasers over fluorescence sensors is their high spectral resolution, which can improve measurement accuracy.

**Figure 3:**
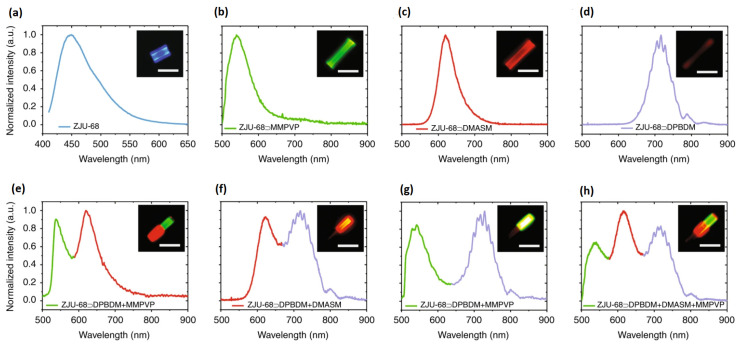
Fluorescence in microcrystals in the visible region [34]. (**a**) ZJU-68. (**b**) ZJU-68/MMPVP. (**c**) ZJU-DMASM. (**d**) ZJU-68/DPBDM. (**e**) ZJU-68/DPBDM + MMPVP. (**f**) ZJU-68/DPBDM + DMASM. (**g**) ZJU-68/DPBDM + MMPVP. (**h**) ZJU-68/DPBDM + DMASM + MMPVP.

Alternatively, solid-state dye lasers consist of host materials such as polymers or composites doped with organic dyes [42,43]. The structure of the host material can significantly impact dye light emission. For example, He et al. [34] reported a microlaser with multiple visible wavelengths in a dye-assembled microcrystal, as shown in Figure 3.

As shown in Figure 3, light emission from microcrystals can be controlled by dyes, including red, green, and blue fluorescence (RGB). The combination of these colors could result in any visible color, which would be helpful in the interaction with certain biological tissues.

### 2.2. Rare Earth Ions

**Figure 4:**
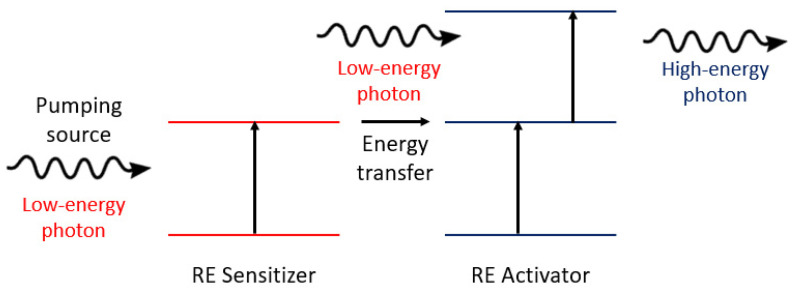
Upconversion process in RE-based lasers.

Rare-earth (RE) ions are efficient gain media for lasing action. A RE-based gain medium requires a host material, typically a crystal such as yttrium aluminum garnet (YAG) or optical fiber. Some examples of conventional RE-based lasers include Nd: YAG, Er: YAG, and ytterbium-doped fiber lasers (YDFLs) [44,45]. In this type of laser, the lasing mechanism is based on energy transitions of trivalent lanthanide ions, which favors the population inversion required for light amplification. In the last few years, the use of RE in microlasers has drawn significant attention due to their inherent properties, such as high stability, wide emission range, and low-cost fabrication [46]. Diverse approaches have been studied to produce microlasers based on RE. For example, Ouyang et al. [47] studied a cost-effective photopolymerization method for fabricating Yb^3+^-doped silica glass with Al and P additions to improve the solubility of RE ions. The doped silica glass was processed to produce a microsphere that served as a gain medium and optical cavity. The microsphere pumped by an optical fiber exhibited lasing emissions from 1033 nm to 1040 nm. Yb^3+^ has been extensively used in fiber lasers due to its low quantum defect, which significantly reduces heat generation. Thermal efficiency is fundamental in biological sensing. Jiang et al. [48] reported a white microlaser incorporating ytterbium, erbium, and thulium in a microsphere. This laser used an upconversion mechanism to generate high-quality red, green, and blue emissions, enabling white lasing. The upconversion process occurs when an active medium absorbs two or more lower-energy photons to emit a higher-energy photon, as shown in Figure 4. RE ions favor the light-emission mechanism due to their multiple metastable energy levels.

Shang et al. [49] reviewed recent developments in lanthanide microlasers based on the upconversion process. According to their study, the typical rare-earth gain media are Yb, Tm, Lu, Gd, Ce, and Er with optical pumping commonly at 980 nm and lasing emission in the UV, visible and near-infrared regions. The gain medium consists of photon upconversion nanocrystals such as NaYF_4_: Yb^3+^Tm^3+^ in different microcavities [50]. One important consideration is that several commercial models of 980 nm laser diodes are available; hence, the practical implementation of these systems outside photonic laboratories is feasible. Among some applications of RE microlasers in biophotonics are imaging, diagnosis, and therapy [51].

### 2.3. Semiconductors

A conventional semiconductor laser is based on a diode structure with a p–n junction. The most common materials used as gain media for lasing emission are direct-gap semiconductors from groups III and V [52]. Their main characteristics are broad wavelength emission (UV-Vis-IR), high electrical-to-optical power efficiency, and compactness. In the last few years, the design of microlasers based on semiconductors has gained attention. Some semiconductors, such as gallium-based materials, have been widely studied given their optoelectronic properties [53,54]. Ma et al. [55] reported a GaN-based microlaser array designed for the fabrication of photonic integrated circuits (PICs). In this approach, microlasers, consisting of a microdisk structure, can be tuned over a wide range in the visible region and exhibit a high-quality factor. Cadmium sulfide (CdS) is a luminescent semiconductor with a band gap of 2.42 eV. Due to its optical properties, CdS has been employed in optoelectronics, photocatalysis, and solar cells [56]. Recently, a CdS nanowire biolaser was reported to monitor the movement of individual cells [57]. The lasing emission from microcavities allowed for the in vivo observation of retinal cells. Table 2 shows the semiconductors used in recent years to produce microlasers.

Recently, other attractive alternatives for gain media, for example, halide perovskite and quantum dot semiconductors, have captured attention due to their optical properties, such as band gap tunability and high photoluminescence quantum yield. Perovskite materials are characterized by the same cubic structure as calcium titanium oxide (CaTiO_3_) [66]. In addition to the structure, perovskite consists of a large cation (A), a small cation (B), and an anion (X), which conventionally is oxygen. However, replacing oxygen with a halide from group VII can enhance the optical properties. In recent years, halide perovskite semiconductors have been studied mainly for their potential to develop photonic devices such as solar cells, photodetectors, LEDs, and lasers [67,68,69,70]. In perovskite semiconductor lasers, cations in the gain medium can be organic, inorganic, or hybrid. These variants enable emission at different wavelengths in the visible region due to the capability of tuning the band-gap energy [67]. One approach to miniaturizing perovskite semiconductor lasers is the use of nanowires. These nanostructures have diameters ranging from 2 nm to 200 nm and lengths in the order of micrometers [71]. Nanowires are usually fabricated using the bottom–up growth approach through the vapor–liquid–solid (VLS) method. Recently, the perovskite CsPbBr_3_ has drawn research interest among gain media based on semiconductors due to its high defect tolerance, which represents an advantage in optoelectronic properties [72]. Tang et al. [73] reported a single-crystalline CsPbBr_3_ perovskite microlaser. The work was based on patterning a polymer microdisk over perovskite to induce a Bound State in the Continuum (BIC) by destructive interference, resulting in strong light confinement. This configuration exhibited an ultra-high Q-factor of 1.04 × 10^5^. This value represents a considerable advantage over other semiconductor-based microlasers.

Alternatively, quantum dot semiconductors consist of particles at the nanoscale, which are typically 10 nm or less. Due to their very small size, these nanomaterials exhibit quantum confinement in these dimensions. This effect produces changes in the electrical and optical properties, for example, in the optical band gap, that could favor the development of tunable optical gain media for lasers. Li et al. [74] designed an array of microlasers based on colloidal quantum dots. The work reported a quasisuperlattice consisting of microrings with lasing emissions in red, green, and blue. In addition to the laser-tuning capability shown in the study, the authors also reported a high-quality factor and low lasing threshold which make it suitable for biophotonic applications. Other studies based on microring quantum dot lasers have reported single-mode infrared emission with thermal tunability [75], multi-wavelength InAs/GaAs lasing emission on silicon [76], and electrically driven microlasers [77,78]. Chang et al. [58] reported quantum dot microlasers with wide tunability in the visible region but with a different approach. In this case, colloidal quantum dot-assembled microspheres, consisting of CdSe/ZnS, were used as the gain medium. One advantage of this work is the significant reduction in the photoluminescence quenching effect and the improved optical stability at high temperatures. A similar work reported luminescent microspheres doped with semiconductor nanocrystals [79]. In this case, the fluorescent material was AgInS_2_/ZnS. This low-toxicity material is water-soluble; hence, it could be used for biosensing purposes. Carbon quantum dots exhibit comparable characteristics. Therefore, their study could contribute to the development of optical microsensing devices. Recently, carbon quantum dots-decorated microspheres were reported as molecular sensors [80]. From these works based on semiconducting quantum dots, it is clear that these materials represent a viable option for the development of microlasers. In addition to laser structures based on microrings and microspheres, another design that has been studied in quantum dot lasers is the microdisk structure [81,82,83]. For example, Luo et al. [84] reported an on-chip microlaser with red emission. The work consists of InP quantum dots and a GaAs/Si disk structure. The microdisk laser exhibited a low-lasing threshold of 500 nW in continuous-wave operation and could potentially be integrated into photonic biosensor circuits.

## 3. Optical Microcavities

The optical cavity is a crucial factor in the output beam of any laser system. The most straightforward configuration comprises two parallel mirrors with a gain medium between them, called Fabry–Perot, which has been widely used in traditional lasers and has recently found applications in microlasers [85,86]. However, in addition to established setups, other alternatives, such as whispering gallery mode (WGM)-based microcavities, have been studied in the last few years, specifically for biosensing [87,88,89]. This section reviews recent work on optical cavities for microlasers.

### 3.1. Whispering Gallery Mode-Based Microcavities

**Figure 5:**
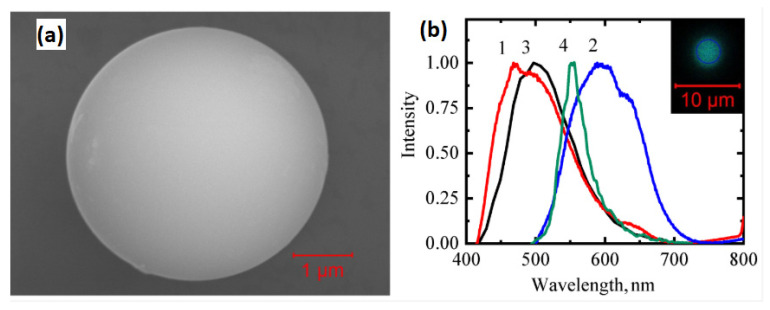
WGM-based microcavity [80]. (**a**) SEM image of a microsphere. (**b**) Photoluminescence spectra of carbon quantum dots (CDs) and quantum dots-doped microspheres (CDMs) with excitation from 405 nm to 488 nm. 1. CDs excited at 405 nm; 2. CDs excited at 488 nm; 3. CDMs excited at 405 nm; 4. CDMs excited at 488 nm.

The whispering gallery concept refers to the effect of confined waves commonly found around a spherical cavity. The English physicist Lord Rayleigh described this phenomenon to explain acoustic waves traveling with low attenuation in St Paul’s Cathedral. In optics, the WGM has been applied to microcavities in optical fibers, microspheres, and microrings (see Figure 5). For example, Peng et al. [90] proposed a WGM scheme based on an exposed-core optical fiber to couple light into microspheres deposited onto the open channel. This microcavity enables high tunability in reflected light, which can be useful in distributed biosensing. In this case, one important contribution is a new coupling scheme that facilitates optical pumping.

**Figure 6:**
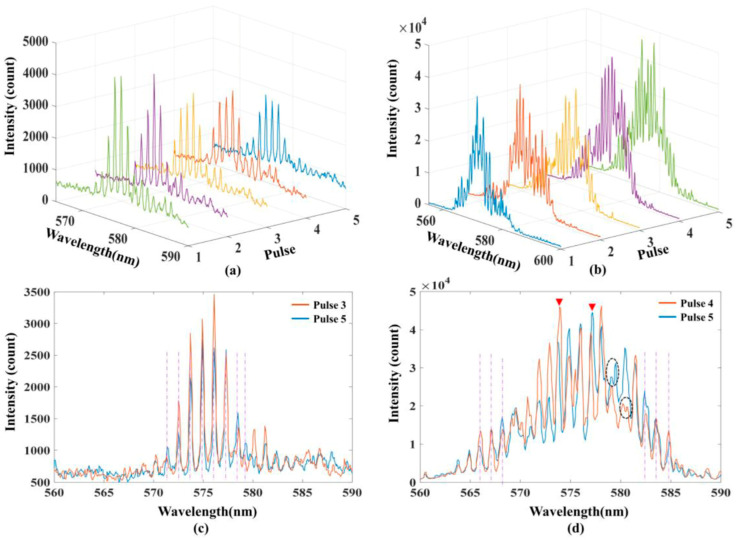
Output spectra in WGM pulsed fiber microlaser [91]. (**a**) Regular WGM emission. (**b**) Random WGM lasing. (**c**) WGM lasing between pulses. (**d**) Random WGM lasing between pulses.

WGM can also be applied in optical fiber microlaser schemes. For example, Cao et al. [91] proposed a WGM fiber microcavity. The experimental setup uses a flexible optical fiber array to modulate the emission with changes in spacing between fibers ranging from 150 µm to 10 µm. The emission in pulsed mode in the visible spectrum is shown in Figure 6. Due to the versatility of sensing techniques in optical fiber sensors, such as interferometry, intensity, or spectral response, WGM-based fiber microcavities could expand their applications in the near future.

**Figure 7:**
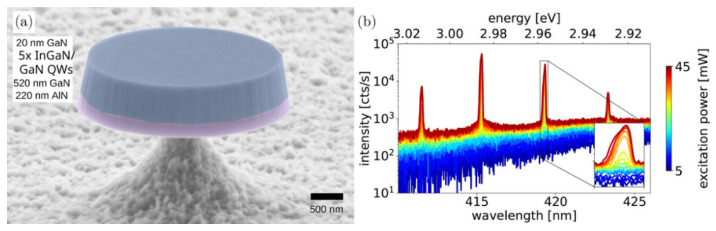
Microdisk laser [92]. (**a**) GaN mushroom-type structure. (**b**) Micro-photoluminescence spectra; inset: visible secondary feature.

Microdisk structures have also been studied using the WGM approach to generate lasing emissions. Typically, in this case, their dimensions range between the nano and micro scales. For instance, Drechsler et al. [92] reported GaN microdisk lasers based on whispering gallery mode resonators (Figure 7). As shown in Figure 7a, the optical cavity consists of a mushroom-type microdisk. The authors studied the same structure with diameters ranging from 3 µm to 6 µm. Figure 7b shows that the microdisk laser exhibited multimode lasing emissions from 410 nm to 425 nm, corresponding to the violet region. These emissions match the optical absorption peaks of hemoglobin [93]; hence, the GaN microdisk laser could be useful for biomarker detection.

A particular type of WGM-based optical microcavity consists of microbubbles. Microbubbles in fluids can be generated under controlled conditions by laser pulses, usually in the order of femtoseconds, for medical purposes and biomedical engineering [94,95]. Microbubbles have also been embedded in optical fiber, resulting in WGM microlasers [96]. One benefit of this kind of device is that it can be easily integrated into a microfluidic chip for biosensing purposes. Among the main advantages of WGM microbubble optical cavities in microlasers are the high quality factor, small form factor, and low threshold [97,98].

### 3.2. Fabry–Perot Microcavities

**Figure 8:**
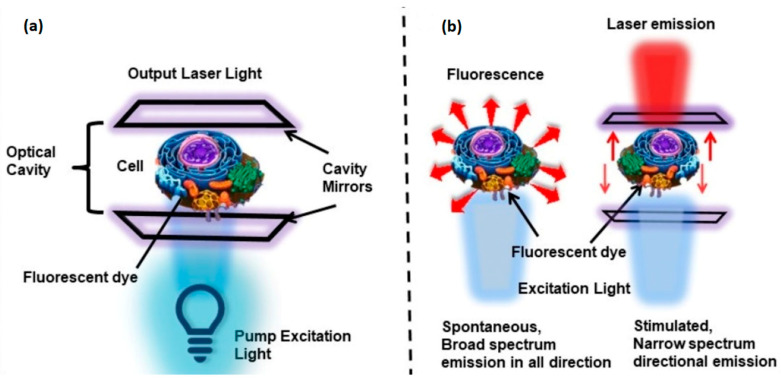
Cell microlaser [41]. (**a**) Cell with fluorescent dyes in a Fabry–Perot cavity. (**b**) Fluorescence vs. laser detection.

A Fabry–Perot cavity consists of two mirrors with the gain medium positioned between them. This configuration has been widely used in traditional lasers. For example, in CO_2_ and Nd: YAG lasers, the optical cavity often consists of a fully and partially reflecting mirror. These mirrors must be accurately aligned to provide efficient light amplification. Commonly, concave mirrors facilitate the alignment of the optical cavity. However, this can be challenging at the micro-scale. Zhang et al. [99] investigated a planar-concave microcavity based on two optical fibers. The ends of both optical fibers were cut at a 90-degree angle to the optical axis. One of the end facets was processed with a CO_2_ laser to create a concave mirror by ablation. A droplet of Coumarine 6 in ethanol was used as the gain medium. The device exhibited very low energy consumption of 25.5 µJ/mm^2^ and lasing emission ranging from 519 nm to 540 nm. Fabry–Perot microcavities have also been studied in solid-state materials. Dang et al. [100] reported a microlaser consisting of rectangular-shaped ZnO microwires. In this case, the laser performance in the UV region was significantly improved with the addition of Ag nanowires. Cell-based microlasers frequently utilize the Fabry–Perot cavity for its simplicity, as shown in Figure 8. As reported in the literature, alignment issues in Fabry–Perot microcavities have been overcome in several laser schemes. This can be attributed to the proximity of the gain medium to the optical cavity.

## 4. Non-Conventional Effects and Configurations

### 4.1. Metal Surface Plasmon

**Figure 9:**
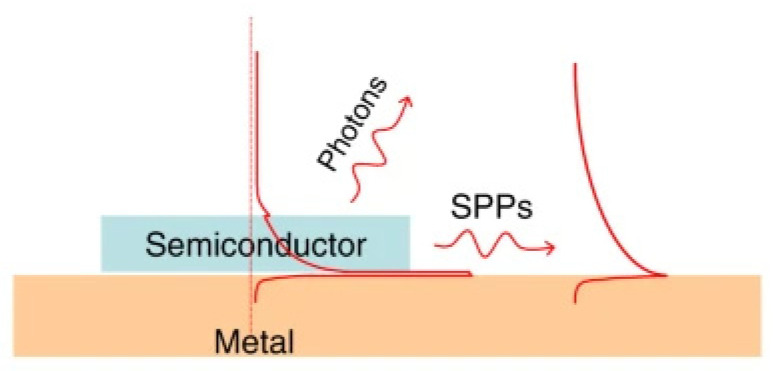
Plasmonic laser structure (spaser) [103].

The plasmonic effect consists of the oscillation of free electrons in the interface of a metal and a dielectric material. This structure creates a waveguide, which allows the formation of an optical cavity. Microlasers based on the plasmonic effect often use a semiconductor as the gain medium. One of the main characteristics of the surface plasmon amplification by stimulated emission of radiation (spaser) is the subwavelength emission it generates. As shown in Figure 9, two emissions are generated by the spaser: surface plasmon polaritons (SPPs) and photons. SPPs are associated with an electromagnetic excitation on the metallic material’s surface [101]. This electromagnetic field decays exponentially with the distance to the surface. Photon emission depends on a semiconductor’s optical properties. The typical wavelength range is in the visible and near-infrared regions. Recently, Guo et al. [102] reported a plasmon-assisted microlaser utilizing a lanthanide-based microcavity. This laser benefits from upconversion, resulting in multiple lasing emissions; hence, it could be used in optoelectronics and high-accuracy sensors.

### 4.2. Random Laser

**Figure 10:**
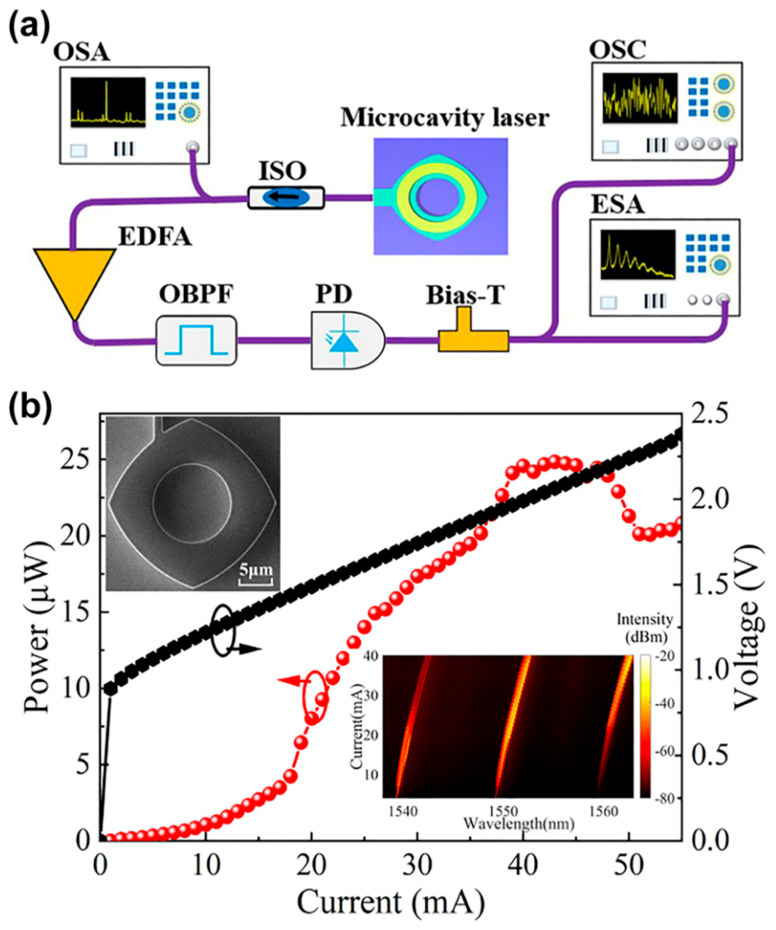
Random microlaser [105]. (**a**) Experimental setup of a deformed square microcavity. (**b**) Optical power and voltage vs input current.

One type of laser that has garnered attention at the micro-scale is the random laser. In this case, light amplification is produced by multiple scatterings within the active medium rather than by a conventional optical cavity consisting of highly reflecting mirrors. Since light scattering exhibits a chaotic nature, random lasers have been applied in various fields, such as cryptography and biomedical imaging [104]. Li et al. [105] designed and studied a self-chaotic microlaser based on a deformed square microcavity. As shown in Figure 10, the laser signal is amplified by an Erbium-doped Fiber Amplifier (EDFA), providing infrared emissions around 1550 nm. The infrared region is especially useful for biomedical imaging [106].

## 5. Applications in Biophotonics

Biophotonics devices have gained interest in recent years for their accuracy and versatility across applications such as bio-imaging and diagnosis [107]. Microlasers offer inherent advantages over traditional lasers that can be useful in biophotonics. The low-lasing threshold favors their use as biosensors without damaging living organisms such as cells. This section provides a review of the current progress on microlaser applications in biophotonics.

### 5.1. Force and Deformation Sensing

Dalaka et al. [108] developed a force sensor to quantify deformations in tumor spheroids and Drosophila larvae. The system consisted of the relationship between the wavelength shift of lasing emission in dye-doped microdroplets and small deformations induced by forces of the order of nanonewtons. This high-resolution force sensor based on a microlaser could be applied to studies of biomechanical forces related to embryogenesis. Recently, Fang et al. [109] also studied the mechanical properties of an organism. The research was based on biocompatible hollow microlasers to evaluate cellular stress in organoids. In this method, the laser response was associated with nanoscale cellular deformation. Zhang et al. [110] reported a flexible sensor based on an array of microlasers. The device can detect mechanical deformations with high precision. The sensor was used to recognize hand gestures, allowing the development of various applications in augmented reality and healthcare [111]. As reported in the literature, a notable advantage of microlaser-based sensing systems is their high accuracy, which is associated with the inherent characteristics of microlasers such as narrow linewidth and high quality factor.

### 5.2. Biomedical Imaging

One of the most interesting applications of microlasers is found in 3D biomedical imaging. Chen et al. [112] proposed a method called Laser-Emission-based Microscope (LEM). This approach uses a bidimensional scanner to measure the emission of fluorophores bound to antibodies confined between two parallel mirrors, forming a Fabry–Perot resonator. This technique was capable of detecting cancer tissues with a resolution in the order of micrometers. Moreover, LEM was also utilized to study neurons in vitro with high sensitivity. Microscopy and optical spectroscopy are two standard methods in biological studies. However, these methods require at least two different tests. Cowie et al. [113] constructed and tested a fluorescence light sheet microscope with an integrated spectrometer. The device was used to measure the refractive index of agarose and zebrafish samples through biointegrated WGM microlasers. Variations in the refractive index can provide valuable insights into biological processes, such as protein diffusion [114] and concentration levels [115]. Moreover, changes in the refractive index can also show DNA information. Caixeiro et al. [20] reported a DNA hybridization sensor based on a WGM microlaser. The study correlates changes in emissions with DNA structural changes, which could be useful in nucleic acid sensing and advanced diagnostics.

**Figure 11:**
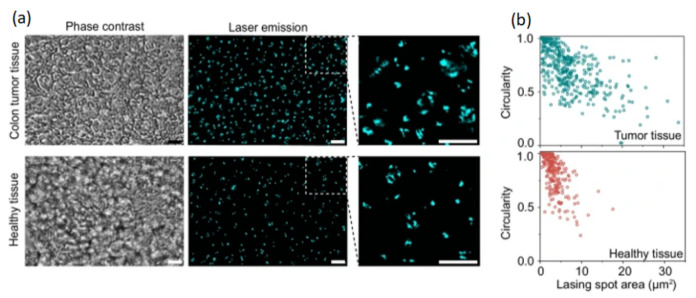
Laser emission from mice tissue [117]. (**a**) Mice colon tumor and healthy tissue (scale bar: 20 µm). (**b**) Correlation between tumor and healthy tissues.

Biomolecular condensates can provide important information related to cells in biotechnology [116]. Typically, their dimensions are in the microscale; thus, they can be used in microlasers. Fang et al. [117] studied a single-cell microlaser for nucleolus differentiation. The laser emission is associated with the biological properties of the cell, such as fluid dynamics and nucleolar size, and could be applied to cancer diagnosis and drug testing. Figure 11 shows cancer detection with a cell laser.

Gong et al. [118] studied self-assembled human amyloid fibril networks through lasing emission in a Fabry–Perot microcavity. It was found that the lasing patterns depend on the structure’s dimension, such as 2D or 3D. These networks are associated with memory and Alzheimer’s disease [119]. Therefore, this type of laser could be a valuable tool for diagnosing neurodegenerative disorders.

### 5.3. In Vivo Sensing

Although most applications of microlasers in biophotonics are based on biolasers, semiconductor microlasers are emerging as a viable alternative in this area. Most laser semiconductors emit at wavelengths below 650 nm, which are highly absorbed or scattered by biological tissues, hence reducing their sensing efficiency. Titze et al. [120] studied a semiconductor microlaser suitable for in vivo sensing applications. In this case, the lasing wavelength was tuned by fabricating heterostructures of AlGaInP potential wells, acting as the amplifier medium, with emissions ranging from 675 to 720 nm. The semiconductor microlaser exhibited low threshold and biocompatibility. According to the study, this laser can be used for bioimaging cell tagging, with applications including studying cancer progression [121] and investigating the effects of drugs [122].

### 5.4. Latest Applications

Due to the growing interest in microlasers for biosensing, several new studies have been reported from late 2025 to early 2026. A bioassay sensor built from several microlasers in a Fabry–Perot cavity was developed by Wu et al. [123]. The microlasers change the laser threshold with analyte concentration. A streptavidin-based model reported a detection limit of 0.1 pg/mL. One advantage of this study is the ability to take multiple measurements simultaneously over a given area, which can provide more information than conventional sensors. Following this work, Wu and Fan [124] also reported a theoretical analysis from the bioassay sensor. Wang et al. [125] reported a liquid molecule microlaser with high sensitivity and low threshold. The laser was effectively used to detect antibody-antigen binding kinetics through changes in lasing spectra. In this case, the main contribution was a significant increase in the sensitivity, which could be extended to other biological applications. Multifunctional sensing is another interesting area in biosensing. A recent work [126] reported a wearable sensor based on a microlaser to measure relative humidity and pH. This work opens new possibilities for non-intrusive biomedical monitoring systems.

## 6. Emerging Approaches: Machine Learning and Digital Sensing

**Figure 12:**
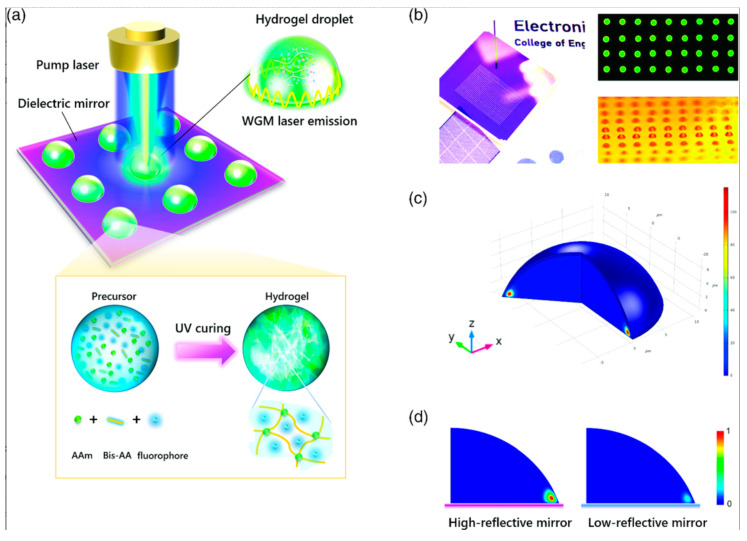
Hydrogel microlaser. (**a**) Hydrogel laser microarray on a dielectric mirror and UV curing process. (**b**) Fluorescent hydrogel array optically pumped by LED. (**c**) Simulation of the electric field distribution of a hydrogel droplet. (**d**) Microdroplet in a high-reflective and low-reflective mirror [131].

In recent years, information technologies in microlasers have gained interest due to their high resolution in biosensing applications. Chan et al. [127] combined machine learning with biolasers to monitor amyloidogenesis. This biological process, associated with protein misfolding, can provide critical information about neurodegenerative diseases such as Parkinson’s and Alzheimer’s. The work consisted of studying dye-based droplet microlasers with a chain of amino acids whose emission wavelength changes in response to biological changes associated with amyloid peptide folding. The small spectral changes were evaluated using deep learning algorithms through a camera using machine vision. The manuscript reported an accuracy of more than 95%, which is suitable for biomedical implementation. Microlaser systems have found promising applications in computational classification tasks. In particular, the study of spiking microlaser neural networks has attracted interest due to their inherent high speed and low energy consumption, which can be useful for photonic chips. Kim et al. [128] developed a machine learning algorithm to predict the dynamics of a microlaser neuron. The work is based on the biomimetic properties of spiking neurons and their similarities with pulsed lasers. In this case, the main application is focused on classification tasks, which could potentially lead to the detection of diseases such as breast cancer or Alzheimer’s [129] with the advantage of improved computational performance. Wu et al. proposed a method to identify tumor cells in blood combining biolasers with deep learning [130]. The work was based on the analysis of nucleic acid-stained cells within a Fabry–Perot optical cavity. A laser emission microscope was used to measure lasing patterns from individual cells. These patterns were processed with a previously unreported Deep Cell-Laser Classifier to detect circulating tumor cells that can contribute information in cancer diagnosis. Gong et al. [131] reported a hydrogel microlaser array to study water–monomer ratios and linkage density (see Figure 12). An advantage of using hydrogel over conventional materials is the high degree of control over functionalizing molecules. The study focused on the analysis of molecular interactions within a microarray consisting of individual microlasers with diameters of approximately 20 µm. Some biomolecules, such as insulin and V5 peptide, were studied, and a correlation was found between their biological structures and the lasing output. One particular advantage of this work is that the laser microarray was produced using a commercial printer, which opens the possibility for related work.

An emerging approach in biophotonics is digital lasing. This method combines laser systems with 2D data, providing additional information at the output. Recently, Zhou et al. [132] designed a microwell array to measure tumor cell concentration. The work consisted of several modified dye-doped WGM microlasers with a 21 µm diameter. Results showed that using a bidimensional 3 × 3 array of microlasers placed in the designed microwell, it was possible to quantify the concentration of particles associated with tumor spheroid-derived samples. This process significantly reduces the time required for result analysis, since the information can be visualized by CCD cameras.

Another interesting digital approach used in biodetection is 3D printing. Wang et al. [133] reported a 3D-printed polymer WGM microlaser for immunoglobulin G (IgG) detection. The laser exhibited a high quality factor and low lasing threshold, which favor biological applications. In particular, the high sensitivity detection of IgG could be used in biomarkers for disease diagnostics of viral or bacterial infections. Reynoso et al. [134] also used 3D printing, but in this case, it was not directly in the active medium but rather to manufacture a phase-array acoustic levitator. The device allows the trapping of dye-doped droplets in the air, which can be used as microlasers. According to the authors of that work, optical parameters such as Q-factors and lasing thresholds are comparable to those in chip devices. This technique could be used for contactless procedures in the field of biophotonics. Cost and complexity are among the main constraints for the 3D manufacturing of microlasers. Recently, a low-cost direct laser writing (DLW) method was utilized to manufacture polymer microstructures coated with rhodamine 6G [135]. These structures, called micropedestals, with dimensions of about 40 µm, exhibited lasing emission in the visible range below 600 nm and a quality factor surpassing 2 × 10^3^. The fabrication of various structures via laser writing is expected in the next few years.

## 7. Discussion and Perspectives

Currently, considerable efforts are being made to investigate the characteristics of microlasers and their applications across various domains of biophotonics, particularly in biomedical imaging and sensing. One of the main areas explored is the analysis of optical materials suitable as gain media. Despite a wide range of options, including rare earths [136,137,138], semiconductors [62,139,140,141], and organic dyes [23,142,143], among others, a critical consideration is their biocompatibility, since most sensing methods rely on microlaser interaction with biological materials. Therefore, in addition to optical performance, recent work has focused on biomaterials. For example, Ta et al. [144] studied a dye-doped polyvinyl alcohol (PVA) as a viable option to implement a biodegradable microlaser. In addition to the potential use of the PVA-based microlaser as an implantable device, the WGM mechanism exhibits a high Q-factor of about 3 × 10^3^ and a low lasing threshold, which favor the efficiency in terms of energy. A recent work [32] demonstrated the feasibility of creating microlasers from only edible substances, including the gain medium and the optical cavity. This study opens the possibility of new biophotonic sensors based on food, for example, for measuring sugar concentration or pH. Fluorescent proteins are also viable options for light amplification in biophotonic microdevices [145,146]. Although fluorescent proteins exhibit limitations, such as low photostability and photobleaching, recent studies have shown advances in these optical parameters [147]. In contrast, conventional semiconductors may not be recognized for their high biocompatibility. However, a recent study on carbon dots with semiconductor properties and improved biocompatibility investigated their use in microlasers [148]. More studies in this direction are expected in the following years.

In recent years, 2D optical materials with advanced properties suitable for microlasers have been developed. MXenes are a class of two-dimensional materials composed of metal carbonitrides, nitrides, and carbides with excellent electrical and optical characteristics [149]. Over the past few years, MXenes have attracted attention as saturable absorbers in pulsed fiber lasers [150,151]. Recently, an MXene-functionalized microresonator sensor for the detection of lead ions was demonstrated [152]. However, the use of MXenes as gain media for microlasers remains a challenge. The main limiting factor for these materials in light emission is their small theoretical band gap [153]. This issue has been overcome with MXene quantum dots [154]. Lin et al. [155] reviewed fluorescent MXene quantum dots and nanosheets with potential applications in biosensing given their high water-interaction and biocompatibility. One advantage of MXenes over organic dyes currently used in microlasers is their resistance to photobleaching [156]. Given their prior use in microresonators and luminescent properties, MXene quantum dots are considered a promising material for microlasers.

One important parameter in lasers is the emission wavelength. Nowadays, microlasers cover the three optical regions, UV, visible, and IR, using different optical materials, which favor their application in biological sensing. However, lasing over a wide wavelength range with a single amplifier medium poses a challenge for microlasers. Recently, Zhao et al. [157] reported multicolor Raman microlasers with a very high Q-factor (>6 × 10^6^) and a low pumping threshold (620 µW). The experimental setup is based on thin-film lithium niobate (TFLN) microcavities. The Raman laser exhibited lasing emission in the visible (406 and 533 nm) and IR regions (813 and 1712 nm). More Raman microlasers are expected in the near future.

Laser miniaturization to its physical limits is another interesting research area. One approach studied for this purpose is the use of surface plasmons. Cho et al. [158] reported a nanolaser based on the plasmonic effect. The work consisted of diffraction-limited gold-coated semiconductor emitters with dimensions around 200 nm. These nanolasers can interact with biological cells, potentially expanding their biophotonic applications.

The biointegration is a critical factor in microlaser applications. Thomson et al. [24] provided an in-depth review of current trends in this field. Biointegration refers to the incorporation of biological materials and structures into the microlaser. The general idea is to acquire biological information by studying lasing emission. Although complete biointegration remains an open challenge, many efforts focus on combining typical lasing materials with cells to develop biosensors or on using fluorescent proteins in optical cavities.

The use of machine learning (ML), algorithms, and artificial intelligence (AI) in designing optical materials suitable for light amplification and digital sensing approaches based on microlasers is expected to increase in the next few years. For example, recent studies on the evolution of fluorescent proteins with green and yellow emissions and on the design of other fluorophores using ML and AI have been reported [159,160,161]. ML can potentially accelerate the development of optical materials with luminescent properties [162,163]. However, currently, there are no works in the literature on the computational design of microlasers based on ML that consider the gain medium, optical cavity, and pump source. The use of ML specifically for microlaser design could accelerate their practical implementation in biophotonics. Neural networks are another promising approach for image classification in microlaser biosensing. Recent works using spiking neurons and genetic algorithms have demonstrated their viability and potential improvements in terms of speed and accuracy [128,164].

Autonomous systems in research are another area that could broaden the development of new technologies based on microlasers. To date, there are some related works on automating research on some materials that could be used as gain media. However, the development of microlasers using autonomous systems such as self-driving laboratories [165] has not been reported. The development of autonomous hardware and software laboratories represents an opportunity to accelerate microlaser research in the near future.

## 8. Conclusions

The study of microlasers has garnered attention in recent years due to their potential applications in various areas of biophotonics, including biomedical imaging and digital sensing. Optical materials play a key role in the development of biocompatible microlasers. The use of fiber-optic fluid lasers based on fluorescent dyes such as rhodamine represents a viable option for biological applications, including DNA studies. One important limitation of organic dyes is the fluorescence degradation caused by photobleaching. Although this disadvantage does not hinder their use in bioapplications, the reproducibility could be a concern. Regarding semiconductors, these may be the most popular materials since they have been widely used in several optoelectronic devices. In particular, semiconducting quantum dots have attracted attention due to their luminescent properties and tunable emission wavelengths. Although they have not traditionally been considered as biomaterials, there are promising recent investigations in this direction. For example, heterostructures consisting of AlGaInP with visible emission have demonstrated biocompatibility and could be used to monitor cancer progression. Moreover, polymers such as PVA have been proposed for encapsulating fluorescent materials to enhance their compatibility with living systems. MXenes quantum dots have also gained attention in recent years for their favorable optical properties for gain media. However, more studies are required to test their performance in microlasers. With respect to rare earths, Yb^3+^ is one of the most popular materials due to its high optical efficiency in lasers. Several optical microcavities have been explored, including Fabry–Perot and WGM-based cavities. The latter are often utilized in microsphere or microring configurations mainly for sensing and detection in biophotonics. Interrogation methods in microlaser-based systems have evolved over the last few years from single-lasing studies to multiple-laser arrays analyzed by machine learning and neural networks with inherent computational advantages such as high speed and strong reliability. Among the most recent bioapplications of microlasers are biosensors, biomedical imaging, cancer detection, DNA refractive index measurements, Alzheimer’s diagnosis, and biomarker detection. More studies related to microcavities based on biocompatible materials and on new approaches built upon digital sensing are expected in the next few years.

## Figures and Tables

**Table 1:** Organic dyes commonly used in laser gain media for microlasers.

Gain Medium	Description	Reference
Rhodamine 640	Dye-doped hollow fiber	[29]
AnBPin	Microcrystal	[30]
DCJTB	PMMA fibers and AU NPs	[31]
Chlorophyll	Dye-doped droplets	[32]
R6G	Microfluidic fiber	[33]
MMPVP-DMASM-DPBDM	Microcrystal	[34]
ASCPI	Microparticles	[35]
Uranin	Full-color textiles	[36]
Bis-MSB	Polymer optical fiber	[37]

**Table 2:** Semiconducting materials, including perovskite and quantum dots used in microlasers.

Semiconductor	Characteristics	Reference
CdSe/ZnS	Colloidal quantum dots (CQD). Ultrastable at high temperatures.	[58]
InAs/GaAs	Quantum dots. Heterostructure on silicon substrate microdisks.	[59]
GaN	Plasmonic nanowire on SiO_2_/Al substrate. UV lasing.	[60]
ZnO/GaN	Hexagonal microrod. Electrically pumped.	[61]
ZnO/In	Microwire electrically pumped.	[62]
AlGaInAs/InP	Multiple quantum wells. Chaotic lasing.	[63]
CdSe/CdZnS	Colloidal quantum well supraparticles.	[64]
IHP	Inorganic halide perovskite. Wave-chaotic lasing.	[65]

## Data Availability

No new data were created or analyzed in this study.

## References

[1] Liu J.M., Chen H.F., Tang S. (2001). Optical-Communication Systems Based on Chaos in Semiconductor Lasers. IEEE Trans. Circuits Syst. I.

[2] Liu H., Sun Z., Chen Y., Zhang W., Chen X., Wong C.-P. (2022). Laser Processing of Flexible In-Plane Micro-Supercapacitors: Progresses in Advanced Manufacturing of Nanostructured Electrodes. ACS Nano.

[3] Pan T., Lu D., Xin H., Li B. (2021). Biophotonic Probes for Bio-Detection and Imaging. Light Sci. Appl..

[4] Kwaśny M., Bombalska A. (2022). Applications of Laser-Induced Fluorescence in Medicine. Sensors.

[5] Latz C., Asshauer T., Rathjen C., Mirshahi A. (2021). Femtosecond-Laser Assisted Surgery of the Eye: Overview and Impact of the Low-Energy Concept. Micromachines.

[6] Pluthero F.G., Kahr W.H.A. (2023). Evaluation of Human Platelet Granules by Structured Illumination Laser Fluorescence Microscopy. Platelets.

[7] Atta D., Elarif A., Al Bahrawy M. (2023). Reactive Oxygen Species Creation by Laser-Irradiated Indocyanine Green as Photodynamic Therapy Modality: An in Vitro Study. Lasers Med. Sci..

[8] Hammerich L., Shevchenko Y., Knorr J., Werner W., Bruneau A., Tacke F. (2023). Resolving 31 Colors on a Standard 3-laser Full Spectrum Flow Cytometer for Immune Monitoring of Human Blood Samples. Cytom. Part B Clin..

[9] Li D., Zhou L., Yu Q., Pu X., Sun Y., Zhou Q., Zhang Y. (2022). Optical Fiber Optofluidic Laser Based on Surfactant Solubilization of Rhodamine B Gain in an Aqueous Solution. Opt. Express.

[10] Tessitore G., Mandl G.A., Maurizio S.L., Kaur M., Capobianco J.A. (2023). The Role of Lanthanide Luminescence in Advancing Technology. RSC Adv..

[11] Ahn N., Livache C., Pinchetti V., Klimov V.I. (2023). Colloidal Semiconductor Nanocrystal Lasers and Laser Diodes. Chem. Rev..

[12] Yu H., Su X., Pan Y., Gao D., Wang J., Chen R., Zhang J., Dou F., Zhang X., Ge K. (2022). Narrow Linewidth CsPbBr3 Perovskite Quantum Dots Microsphere Lasers. Opt. Mater..

[13] Jiang S., Guo C., Che K., Luo Z., Du T., Fu H., Xu H., Cai Z. (2019). Visible Raman and Brillouin Lasers from a Microresonator/ZBLAN-Fiber Hybrid System. Photonics Res..

[14] Yuyama K., Kawaguchi H., Wei R., Omatsu T. (2023). Fabrication of an Array of Hemispherical Microlasers Using Optical Vortex Laser-Induced Forward Transfer. ACS Photonics.

[15] Caixeiro S., Kunstmann-Olsen C., Schubert M., Hill J., Barnard I.R.M., Simmons M.D., Johnson S., Gather M.C. (2023). Local Sensing of Absolute Refractive Index During Protein-Binding Using Microlasers with Spectral Encoding. Adv. Opt. Mater..

[16] Zhou X., Gather M.C., Scarcelli G. (2024). High-Sensitivity Detection of Changes in Local Refractive Index and Absorption by Analyzing WGM Microlaser Emission via a 2D Dispersion Spectrometer. ACS Photonics.

[17] Liu M., Jiang M., Zhou X., Kan C., Shi D. (2022). Performance-Enhanced Single-Mode Microlasers in an Individual Microwire Covered by Ag Nanowires. Opt. Laser Technol..

[18] Frigenti G., Berneschi S., Farnesi D., Pelli S., Righini G.C., Soria S., Dumeige Y., Féron P., Ristić D., Prudenzano F. (2023). Rare Earth-Doped Glass Whispering Gallery Mode Micro-Lasers. Eur. Phys. J. Plus.

[19] Zhukov A.E., Kryzhanovskaya N.V., Moiseev E.I., Maximov M.V. (2021). Quantum-Dot Microlasers Based on Whispering Gallery Mode Resonators. Light Sci. Appl..

[20] Caixeiro S., Dörrenhaus R., Popczyk A., Schubert M., Kath-Schorr S., Gather M.C. (2025). DNA Sensing with Whispering Gallery Mode Microlasers. Nano Lett..

[21] Feng Z., Bai L. (2018). Advances of Optofluidic Microcavities for Microlasers and Biosensors. Micromachines.

[22] Toropov N., Cabello G., Serrano M.P., Gutha R.R., Rafti M., Vollmer F. (2021). Review of Biosensing with Whispering-Gallery Mode Lasers. Light Sci. Appl..

[23] Qiao Z., Sun H., Chen Y.-C. (2024). Droplet Microlasers: From Fundamentals to Multifunctional Applications. Appl. Phys. Rev..

[24] Thomson C.A., Popczyk A., Schubert M., Gather M.C. (2025). Biointegrated Microlasers: Technologies, Applications, and Emerging Developments. Optica.

[25] Zhou Z., Chen D., Fan R., Li X., Chen Z., Luo T., Dong Z., Jiang Y. (2020). 500 Hz Ultraviolet Dye Laser with Pulse Energy 1.7 mJ and Potential for PLIF Imaging. J. Mod. Opt..

[26] Avellanal-Zaballa E., Gartzia-Rivero L., Bañuelos J., García-Moreno I., R. Agarrabeitia A., Peña-Cabrera E., Ortiz M.J. (2020). A Palette of Efficient and Stable Far-Red and NIR Dye Lasers. Appl. Sci..

[27] Kim M.M., Darafsheh A. (2020). Light Sources and Dosimetry Techniques for Photodynamic Therapy. Photochem. Photobiol..

[28] Dai D.P., Xia Y., Yin Y.N., Yang X.X., Fang Y.F., Li X.J., Yin J.P. (2014). A Linewidth-Narrowed and Frequency-Stabilized Dye Laser for Application in Laser Cooling of Molecules. Opt. Express.

[29] Anugop B., Anand V.R., Kailasnath M. (2023). Amplification of Whispering Gallery Microlaser Emission Using Dye-Doped Graded-Index Polymer Optical Fiber. Opt. Commun..

[30] Sun C., Li J., Ye J., Chai Y., Ding Y., Ma J., Wang L., Tian Y., Zhang H. (2025). Mechanochromic Organic Micro-Laser. Angew. Chem. Int. Ed..

[31] Shi B., Zhang Y., Lv H., Ma L., Zhang S., Wang M., Wang X. (2024). A Micro Random Laser of Dye Solution-Filled Tube System Based on Electrospun Fibers. J. Lumin..

[32] Anwar A.R., Mur M., Michailidou G., Bikiaris D.N., Humar M. (2025). Microlasers Made Entirely from Edible Substances. Adv. Opt. Mater..

[33] Zhang H., Han B., Li X., Zhao Y., Zhang Y.-N. (2024). An Optical Fiber Optofluidic Laser Biosensor for Rapid Hemoglobin Detection Using Organic Dye. J. Light. Technol..

[34] He H., Cui Y., Li H., Shao K., Chen B., Qian G. (2020). Controllable Broadband Multicolour Single-Mode Polarized Laser in a Dye-Assembled Homoepitaxial MOF Microcrystal. Light Sci. Appl..

[35] Song F., Zhang C., Dong H., Fan Y., Wu M.-Y., Shan G., Lai P., Gao H., Zhao Y.S., Chen S. (2021). A Switchable Multimode Microlaser Based on an AIE Microsphere. J. Mater. Chem. C.

[36] Ruan J., Guo D., Niu B., Ge K., Zhai T. (2022). Whispering-Gallery-Mode Full-Color Laser Textiles and Their Anticounterfeiting Applications. NPG Asia Mater..

[37] Anugop B., Simon J., Madanan K. (2023). Blue Micro-Lasing from Dye-Doped Hollow Polymer Optical Fiber with an Ag Nanoparticle-Doped Microring. Appl. Opt..

[38] Yang X., Gong C., Liu Y., Rao Y., Smietana M., Gong Y. (2021). Recent Progress in Fiber Optofluidic Lasing and Sensing. Photonic Sens..

[39] Parker H.E., Sengupta S., Harish A.V., Soares R.R.G., Joensson H.N., Margulis W., Russom A., Laurell F. (2022). A Lab-in-a-Fiber Optofluidic Device Using Droplet Microfluidics and Laser-Induced Fluorescence for Virus Detection. Sci. Rep..

[40] Pendão C., Silva I. (2022). Optical Fiber Sensors and Sensing Networks: Overview of the Main Principles and Applications. Sensors.

[41] Sarbadhikary P., George B.P., Abrahamse H. (2022). Paradigm Shift in Future Biophotonics for Imaging and Therapy: Miniature Living Lasers to Cellular Scale Optoelectronics. Theranostics.

[42] García-Villarreal A., Robledo-Martinez A., Espejo-Ramos H., Sobral H. (2021). Characterization of a Pulsed Rhodamine/Ethanol Dye Laser. J. Phys. Conf. Ser..

[43] Stefańska D., Suski M., Zygmunt A., Stachera J., Furmann B. (2019). Tunable Single-Mode Cw Energy-Transfer Dye Laser Directly Optically Pumped by a Diode Laser. Opt. Laser Technol..

[44] Estrin N.E., Moraschini V., Zhang Y., Romanos G.E., Sculean A., Miron R.J. (2022). Combination of Nd:YAG and Er:YAG Lasers in Non-Surgical Periodontal Therapy: A Systematic Review of Randomized Clinical Studies. Lasers Med. Sci..

[45] Zeng L., Wang X., Ye Y., Wang L., Yang B., Xi X., Wang P., Pan Z., Zhang H., Shi C. (2023). High Power Ytterbium-Doped Fiber Lasers Employing Longitudinal Vary Core Diameter Active Fibers. Photonics.

[46] Chen Z., Dong G., Barillaro G., Qiu J., Yang Z. (2021). Emerging and Perspectives in Microlasers Based on Rare-Earth Ions Activated Micro-/Nanomaterials. Prog. Mater. Sci..

[47] Ouyang M., Wu J., Huang X., Dong G. (2024). Enhanced Rare-Earth Ion Doping in Silica Glass by Photopolymerization for Microlaser. J. Lumin..

[48] Jiang B., Zhu S., Wang W., Li J., Dong C.-H., Shi L., Zhang X. (2022). Room-Temperature Continuous-Wave Upconversion White Microlaser Using a Rare-Earth-Doped Microcavity. ACS Photonics.

[49] Shang Y., Chen T., Ma T., Hao S., Lv W., Jia D., Yang C. (2022). Advanced Lanthanide Doped Upconversion Nanomaterials for Lasing Emission. J. Rare Earths.

[50] Liu Y., Teitelboim A., Fernandez-Bravo A., Yao K., Altoe M.V.P., Aloni S., Zhang C., Cohen B.E., Schuck P.J., Chan E.M. (2020). Controlled Assembly of Upconverting Nanoparticles for Low-Threshold Microlasers and Their Imaging in Scattering Media. ACS Nano.

[51] Ding S., Lu L., Fan Y., Zhang F. (2020). Recent Progress in NIR-II Emitting Lanthanide-Based Nanoparticles and Their Biological Applications. J. Rare Earths.

[52] Tournié E., Monge Bartolome L., Rio Calvo M., Loghmari Z., Díaz-Thomas D.A., Teissier R., Baranov A.N., Cerutti L., Rodriguez J.-B. (2022). Mid-Infrared III–V Semiconductor Lasers Epitaxially Grown on Si Substrates. Light Sci. Appl..

[53] Adewale A.A., Yahaya A.A., Agbolade L.O., Yusuff O.K., Azeez S.O., Babalola K.K., Suleman K.O., Sanusi Y.K., Chik A. (2024). Optoelectronic and Mechanical Properties of Gallium Arsenide Alloys: Based on Density Functional Theory. Chem. Phys. Impact.

[54] Hu Z., Hu S., Zhang M., Wu W., Fan S., Su J. (2025). The Optoelectronic Synergistic Properties Based on Indium–Gallium–Zinc Oxide Neuromorphic Transistors. Nanotechnology.

[55] Ma L., Zhong H., Yang T., Ying L., Chen J., Su Z., Chen S., Weng G., Mei Y., Zhang B. (2025). On-Chip Broadband Multiwavelength Microlaser Array in Visible Region. Laser Photonics Rev..

[56] Ismail W., Ibrahim G., Habib M.A., Alduaij O.K., Abdelfatah M., El-Shaer A. (2023). Advancement of Physical and Photoelectrochemical Properties of Nanostructured CdS Thin Films toward Optoelectronic Applications. Nanomaterials.

[57] Li X., Zhang W., Li Y., Wu X., Wang M., Tan X., Paulus Y.M., Fan X., Wang X. (2022). In Vivo Tracking of Individual Stem Cells Labeled with Nanowire Lasers Using Multimodality Imaging. Biomed. Opt. Express.

[58] Chang H., Zhong Y., Dong H., Wang Z., Xie W., Pan A., Zhang L. (2021). Ultrastable Low-Cost Colloidal Quantum Dot Microlasers of Operative Temperature up to 450 K. Light Sci. Appl..

[59] Zhukov A.E., Kryzhanovskaya N.V., Moiseev E.I., Dragunova A.S., Tang M., Chen S., Liu H., Kulagina M.M., Kadinskaya S.A., Zubov F.I. (2020). InAs/GaAs Quantum Dot Microlasers Formed on Silicon Using Monolithic and Hybrid Integration Methods. Materials.

[60] Zhang Q., Li G., Liu X., Qian F., Li Y., Sum T.C., Lieber C.M., Xiong Q. (2014). A Room Temperature Low-Threshold Ultraviolet Plasmonic Nanolaser. Nat. Commun..

[61] Dai J., Xu C.X., Sun X.W. (2011). ZnO-Microrod/p-GaN Heterostructured Whispering-Gallery-Mode Microlaser Diodes. Adv. Mater..

[62] Xu K., Jiang M., Wan P., Shi D., Kan C. (2026). Advanced Low-Threshold Continuous-Wave Electrical Pumping Ultraviolet Fabry–Pérot Microlaser. J. Mater. Sci. Technol..

[63] Ma C.-G., Xiao J.-L., Xiao Z.-X., Yang Y.-D., Huang Y.-Z. (2022). Chaotic Microlasers Caused by Internal Mode Interaction for Random Number Generation. Light Sci. Appl..

[64] Alves P.U., Quinn G., Strain M.J., Durmusoglu E.G., Sharma M., Demir H.V., Edwards P.R., Martin R.W., Dawson M.D., Laurand N. (2025). Colloidal Semiconductor Quantum Well Supraparticles as Low-Threshold and Photostable Microlasers. Adv. Mater. Technol..

[65] Li F., Wu Y., Chen P., Feng L., Wang Z., Ren Y., Wang Y. (2022). High-Quality Wave-Chaotic Microlasers from Deformed Halide Perovskite Cavities. ACS Photonics.

[66] Zhang L., Mei L., Wang K., Lv Y., Zhang S., Lian Y., Liu X., Ma Z., Xiao G., Liu Q. (2023). Advances in the Application of Perovskite Materials. Nano-Micro Lett..

[67] Zhang Q., Shang Q., Su R., Do T.T.H., Xiong Q. (2021). Halide Perovskite Semiconductor Lasers: Materials, Cavity Design, and Low Threshold. Nano Lett..

[68] Yoo J.J., Seo G., Chua M.R., Park T.G., Lu Y., Rotermund F., Kim Y.-K., Moon C.S., Jeon N.J., Correa-Baena J.-P. (2021). Efficient Perovskite Solar Cells via Improved Carrier Management. Nature.

[69] Li L., Ye S., Qu J., Zhou F., Song J., Shen G. (2021). Recent Advances in Perovskite Photodetectors for Image Sensing. Small.

[70] Yang D., Zhao B., Yang T., Lai R., Lan D., Friend R.H., Di D. (2022). Toward Stable and Efficient Perovskite Light-Emitting Diodes. Adv. Funct. Mater..

[71] Gu Z., Song Q., Xiao S. (2021). Nanowire Waveguides and Lasers: Advances and Opportunities in Photonic Circuits. Front. Chem..

[72] Ma W., Liu L., Qin H., Gao R., He B., Gou S., He Y., Ouyang X. (2023). The Total Ionizing Dose Effects on Perovskite CsPbBr3 Semiconductor Detector. Sensors.

[73] Tang H., Wang Y., Chen Y., Wang K., He X., Huang C., Xiao S., Yu S., Song Q. (2023). Ultrahigh-Q Lead Halide Perovskite Microlasers. Nano Lett..

[74] Li H., Zhao Y., Qiu Y., Gao H., He K., Yang J., Zhao Y., OuYang G., Ma N., Wei X. (2024). Multi-Interfacial Confined Assembly of Colloidal Quantum Dots Quasisuperlattice Microcavities for High-Resolution Full-Color Microlaser Arrays. Adv. Mater..

[75] Zhu S., Ma X., Liu C., Luo W., Liu J., Shi B., Guo W., Lau K.M. (2021). Controlled Single-Mode Emission in Quantum Dot Micro-Lasers. Opt. Express.

[76] Zhu L., Wang J., Yang Y., Wu G., Chen W., Huang Y., Ren X. (2021). Design and Optimization of Unidirectional Emitting Multi-Wavelength InAs/GaAs Quantum Dot Microring Lasers on Silicon. Appl. Phys. A.

[77] Kryzhanovskaya N., Zhukov A., Moiseev E., Maximov M. (2021). III–V Microdisk/Microring Resonators and Injection Microlasers. J. Phys. D Appl. Phys..

[78] Wan Y., Jung D., Shang C., Collins N., MacFarlane I., Norman J., Dumont M., Gossard A.C., Bowers J.E. (2019). Low-Threshold Continuous-Wave Operation of Electrically Pumped 1.55 Μm InAs Quantum Dash Microring Lasers. ACS Photonics.

[79] Kurassova K., Filatov N., Karamysheva S., Bukatin A., Starovoytov A., Vartanyan T., Vollmer F., Toropov N.A. (2024). Microfluidics-Driven Dripping Technique for Fabricating Polymer Microspheres Doped with AgInS_2_ /ZnS Quantum Dots. ACS Omega.

[80] Starovoytov A.A., Soloveva E.O., Kurassova K., Bogdanov K.V., Arefina I.A., Shevchenko N.N., Vartanyan T.A., Dadadzhanov D.R., Toropov N.A. (2024). Carbon Dot-Decorated Polystyrene Microspheres for Whispering-Gallery Mode Biosensing. Photonics.

[81] Zhukov A.E., Kryzhanovskaya N.V., Moiseev E.I., Nadtochiy A.M., Dragunova A.S., Maximov M.V., Zubov F.I., Kadinskaya S.A., Berdnikov Y., Kulagina M.M. (2020). Impact of Self-Heating and Elevated Temperature on Performance of Quantum Dot Microdisk Lasers. IEEE J. Quantum Electron..

[82] Zhou T., Tang M., Xiang G., Fang X., Liu X., Xiang B., Hark S., Martin M., Touraton M.-L., Baron T. (2019). Ultra-Low Threshold InAs/GaAs Quantum Dot Microdisk Lasers on Planar on-Axis Si (001) Substrates. Optica.

[83] Makhov I., Ivanov K., Moiseev E., Dragunova A., Fominykh N., Shernyakov Y., Maximov M., Kryzhanovskaya N., Zhukov A. (2023). Two-State Lasing in Microdisk Laser Diodes with Quantum Dot Active Region. Photonics.

[84] Luo W., Lin L., Huang J., Han Y., Lau K.M. (2021). Red-Emitting InP Quantum Dot Micro-Disk Lasers Epitaxially Grown on (001) Silicon. Opt. Lett..

[85] Xiao Z., Zhu Z., Wang Q., Zhao X., Liu Y., Huang H., Shen Y., Wu X., Wu X., Chen C. (2025). Intracavity Light Field Shaping by Micro-Optics Integrated Inside Fabry-Pérot Microlasers. Laser Photonics Rev..

[86] Testa G., Coviello V., Persichetti G., Bernini R. (2025). High Performance Polymeric Fabry-Pérot Microcavities for Sensing and Lasing Applications. Polymers.

[87] Loyez M., Adolphson M., Liao J., Yang L. (2023). From Whispering Gallery Mode Resonators to Biochemical Sensors. ACS Sens..

[88] Houghton M.C., Kashanian S.V., Derrien T.L., Masuda K., Vollmer F. (2024). Whispering-Gallery Mode Optoplasmonic Microcavities: From Advanced Single-Molecule Sensors and Microlasers to Applications in Synthetic Biology. ACS Photonics.

[89] Yang S., Wang Y., Sun H. (2015). Advances and Prospects for Whispering Gallery Mode Microcavities. Adv. Opt. Mater..

[90] Peng L., Riesen N., Li J., Han M., Nguyen L.V., Ebendorff-Heidepriem H., Warren-Smith S.C. (2021). Whispering Gallery Mode Excitation Using Exposed-Core Fiber. Opt. Express.

[91] Cao B., He Z., Zhang W. (2025). Random Emission and Control of Whispering Gallery Mode Using Flexible Optical Fiber. Photonics.

[92] Drechsler M.L., Choi L.S., Tabataba-Vakili F., Nippert F., Koulas-Simos A., Lorke M., Reitzenstein S., Alloing B., Boucaud P., Wagner M.R. (2024). Multimode Emission in GaN Microdisk Lasers. Laser Photonics Rev..

[93] Taylor-Williams M., Spicer G., Bale G., Bohndiek S.E. (2022). Noninvasive Hemoglobin Sensing and Imaging: Optical Tools for Disease Diagnosis. J. Biomed. Opt..

[94] Rao D.C.K., Mooss V.S., Mishra Y.N., Hanstorp D. (2022). Controlling Bubble Generation by Femtosecond Laser-Induced Filamentation. Sci. Rep..

[95] Yamamoto A., Tamagawa M. (2025). Generation and Control of Underwater Microshock Waves and Microbubbles by a Femtosecond Pulse Laser. Shock Waves.

[96] Sun X., Fang G., Nie N., Yuan Z., Fu B., Zhou T., Tseng P., Chen Y. (2025). Microbubble-Integrated Polymer Fiber Lasers for Multispectral Sensing on Chip. Laser Photonics Rev..

[97] Zhao X., Guo Z., Zhou Y., Guo J., Liu Z., Li Y., Luo M., Wu X. (2022). Optical Whispering-Gallery-Mode Microbubble Sensors. Micromachines.

[98] Babichev A., Makhov I., Kryzhanovskaya N., Troshkov S., Zadiranov Y., Salii Y., Kulagina M., Bobrov M., Vasil’ev A., Blokhin S. (2025). Low-Threshold Surface-Emitting Whispering-Gallery Mode Microlasers. IEEE J. Select. Top. Quantum Electron..

[99] Zhang T., Jia Z., Li Z., Hua S., Chen J., Wang W., Liu S. (2020). Generation of Optofluidic Laser in Stable Fiber Fabry–Pérot Microcavities. Opt. Commun..

[100] Dang H., Zhou X., Li B., Kan C., Jiang M. (2021). Higher-Performance Fabry-Perot Microlaser Enabled by a Quadrilateral Microwire via Ag Nanowires Decoration. Opt. Mater..

[101] Zayats A.V., Smolyaninov I.I., Maradudin A.A. (2005). Nano-Optics of Surface Plasmon Polaritons. Phys. Rep..

[102] Guo L., Ji M., Kang B., Zhang M., Xie X., Wu Z., Chen H., Deckert V., Zhang Z. (2024). Plasmon-Assisted Mode Selection Lasing in a Lanthanide-Based Microcavity. Adv. Photonics.

[103] Azzam S.I., Kildishev A.V., Ma R.-M., Ning C.-Z., Oulton R., Shalaev V.M., Stockman M.I., Xu J.-L., Zhang X. (2020). Ten Years of Spasers and Plasmonic Nanolasers. Light Sci. Appl..

[104] Gomes A.S.L., Moura A.L., De Araújo C.B., Raposo E.P. (2021). Recent Advances and Applications of Random Lasers and Random Fiber Lasers. Prog. Quantum Electron..

[105] Li J.-C., Xiao J.-L., Yang Y.-D., Chen Y.-L., Huang Y.-Z. (2023). Random Bit Generation Based on a Self-Chaotic Microlaser with Enhanced Chaotic Bandwidth. Nanophotonics.

[106] Zhao M., Sik A., Zhang H., Zhang F. (2023). Tailored NIR-II Lanthanide Luminescent Nanocrystals for Improved Biomedical Application. Adv. Opt. Mater..

[107] Chuang S.-C., Yu S.-A., Hung P.-C., Lu H.-T., Nguyen H.-T., Chuang E.-Y. (2023). Biological Photonic Devices Designed for the Purpose of Bio-Imaging with Bio-Diagnosis. Photonics.

[108] Dalaka E., Hill J.S., Booth J.H.H., Popczyk A., Pulver S.R., Gather M.C., Schubert M. (2024). Deformable Microlaser Force Sensing. Light Sci. Appl..

[109] Fang G., Ho B.X., Xu H., Gong C., Qiao Z., Liao Y., Zhu S., Lu H., Nie N., Zhou T. (2024). Compressible Hollow Microlasers in Organoids for High-Throughput and Real-Time Mechanical Screening. ACS Nano.

[110] Zhang C., Dong H., Zhang C., Fan Y., Yao J., Zhao Y.S. (2021). Photonic Skins Based on Flexible Organic Microlaser Arrays. Sci. Adv..

[111] Gupta S., Bagga S., Sharma D.K., Gupta D., Hassanien A.E., Khanna A. (2020). Hand Gesture Recognition for Human Computer Interaction and Its Applications in Virtual Reality. Advanced Computational Intelligence Techniques for Virtual Reality in Healthcare.

[112] Chen Y.-C., Tan X., Sun Q., Chen Q., Wang W., Fan X. (2017). Laser-Emission Imaging of Nuclear Biomarkers for High-Contrast Cancer Screening and Immunodiagnosis. Nat. Biomed. Eng..

[113] Cowie R.C., Schubert M. (2024). Light Sheet Microscope Scanning of Biointegrated Microlasers for Localized Refractive Index Sensing. Photonics Res..

[114] Liang S., Xu J., Weng L., Dai H., Zhang X., Zhang L. (2006). Protein Diffusion in Agarose Hydrogel in Situ Measured by Improved Refractive Index Method. J. Control. Release.

[115] Dai H., Wu J., Wang Y., Tan S., Liang S., Jiang B., Zhao N., Xu J. (2011). Diffusion of Levofloxacin Mesylate in Agarose Hydrogels Monitored by a Refractive-index Method. J. Appl. Polym. Sci..

[116] Erkamp N.A., Verwiel M.A.M., Qian D., Sneideris T., Spaepen F.A., Weitz D.A., Van Hest J.C.M., Knowles T.P.J. (2024). Biomolecular Condensates with Complex Architectures via Controlled Nucleation. Nat. Chem. Eng..

[117] Fang G., Qiao Z., Huang L., Zhu H., Xie J., Zhou T., Xiong Z., Su I.-H., Jin D., Chen Y.-C. (2024). Single-Cell Laser Emitting Cytometry for Label-Free Nucleolus Fingerprinting. Nat. Commun..

[118] Gong C., Qiao Z., Zhu S., Wang W., Chen Y.-C. (2021). Self-Assembled Biophotonic Lasing Network Driven by Amyloid Fibrils in Microcavities. ACS Nano.

[119] Peng W., Zhu Z., Lou J., Chen K., Wu Y., Chang C. (2023). High-Frequency Terahertz Waves Disrupt Alzheimer’s β-Amyloid Fibril Formation. eLight.

[120] Titze V.M., Caixeiro S., Di Falco A., Schubert M., Gather M.C. (2022). Red-Shifted Excitation and Two-Photon Pumping of Biointegrated GaInP/AlGaInP Quantum Well Microlasers. ACS Photonics.

[121] Shu R., Evtimov V.J., Hammett M.V., Nguyen N.-Y.N., Zhuang J., Hudson P.J., Howard M.C., Pupovac A., Trounson A.O., Boyd R.L. (2021). Engineered CAR-T Cells Targeting TAG-72 and CD47 in Ovarian Cancer. Mol. Ther.–Oncolytics.

[122] Tabana Y., Babu D., Fahlman R., Siraki A.G., Barakat K. (2023). Target Identification of Small Molecules: An Overview of the Current Applications in Drug Discovery. BMC Biotechnol..

[123] Wu W., Cao Y., Tan X., Fan X. (2025). Sensitive Bioassay with an Ultralarge Dynamic Range via Microlaser Ensemble Quenching. ACS Sens..

[124] Wu W., Fan X. (2026). Theoretical Analysis of Bioassays Based on Microlaser Ensembles. IEEE J. Select. Top. Quantum Electron..

[125] Wang Y., Hu Y.-H., Wu J.-L., Duan R., Jiao Y.-F., Wang H.-Y., Jiang L.-Y., Kuang L.-M., Sun H.-D., Jing H. (2026). Liquid Photonic-Molecule Microlasers for Ultrasensitive Biosensing. Nat. Commun..

[126] Ruan J., Li Y., Wang K., Fang C., Zhai T. (2026). Flexible Hydrogel Laser Textile for Multifunctional Sensing. Nano Res..

[127] Chan K.K., Shang L.-W., Qiao Z., Liao Y., Kim M., Chen Y.-C. (2022). Monitoring Amyloidogenesis with a 3D Deep-Learning-Guided Biolaser Imaging Array. Nano Lett..

[128] Kim G., Dubernard M., El-Nakouzi S.V., Masominia A.-H., Barbay S., Calvet L.E. (2025). Compact Classification Using the Biomimetic Properties of Ultrafast Spiking Microlaser Neurons. Neuromorph. Comput. Eng..

[129] Sordillo D.C., Sordillo L.A. (2022). The Principles of Machine Learning Algorithms: Applications to Biophotonics and Disease. Biophotonics, Tryptophan and Disease.

[130] Wu W., Zhang Y., Tan X., Chen Y., Cao Y., Sahai V., Peterson N., Goo L., Fry S., Kathawate V. (2025). Antigen-Independent Single-Cell Circulating Tumor Cell Detection Using Deep-Learning-Assisted Biolasers. Biosens. Bioelectron..

[131] Gong X., Qiao Z., Guan P., Feng S., Yuan Z., Huang C., Chang G.-E., Chen Y.-C. (2020). Hydrogel Microlasers for Versatile Biomolecular Analysis Based on a Lasing Microarray. Adv. Photonics Res..

[132] Zhou T., Fang G., Wang Z., Qiao Z., Nie N., Fu B., Tseng P.-H., Sun X., Chen Y.-C. (2025). Digital Lasing Biochip for Tumor-Derived Exosome Analysis. Anal. Chem..

[133] Wang Z., Raza M., Zhou B., Wang N., Krishnaiah K.V., Qin Y., Zhang A.P. (2025). 3D Micro-Printed Polymer Limacon-Shaped Whispering-Gallery-Mode Microlaser Sensors for Label-Free Biodetection. Opt. Lett..

[134] Reynoso-de La Cruz H.M., Hernández-Campos E.D., Ortiz-Ricardo E., Martínez-Borquez A., Rosas-Román I., Contreras V., Ramos-Ortiz G., Mendoza-Santoyo B., Zurita-Lopez C.I., Castro-Beltrán R. (2024). Acoustically Levitated Whispering-Gallery Mode Microlasers. Opt. Laser Technol..

[135] Reynoso-de La Cruz H.M., Ortiz-Ricardo E., Camarena-Chávez V.A., Martínez-Borquez A., Gutiérrez-Juárez G., U’Ren A.B., Castro-Beltrán R. (2021). Low-Cost Fabrication of Microlasers Based on Polymeric Micropedestals. Appl. Opt..

[136] Fan H., Chen X., Fan H., Wang A., Chang R. (2024). Experimental Neodymium-Doped Microlaser with Theoretical Analysis of the Thermo-Optic Effect. J. Opt. Soc. Am. B.

[137] Anashkina E.A. (2020). Laser Sources Based on Rare-Earth Ion Doped Tellurite Glass Fibers and Microspheres. Fibers.

[138] Jiang B., Zhu S., Ren L., Shi L., Zhang X. (2022). Simultaneous Ultraviolet, Visible, and near-Infrared Continuous-Wave Lasing in a Rare-Earth-Doped Microcavity. Adv. Photonics.

[139] Thung Y.T., Duan R., Durmusoglu E.G., He Y., Xiao L., Lee C.X.X., Lew W.S., Zhang L., Demir H.V., Sun H. (2023). Ultrahigh Quality Microlasers from Controlled Self-Assembly of Ultrathin Colloidal Semiconductor Quantum Wells. Laser Photonics Rev..

[140] Liao K., Zhong Y., Du Z., Liu G., Li C., Wu X., Deng C., Lu C., Wang X., Chan C.T. (2023). On-Chip Integrated Exceptional Surface Microlaser. Sci. Adv..

[141] Xu K., Zhou X., Wan P., Shi D., Kan C., Jiang M. (2025). Submicron Ultraviolet Single-Mode Microlaser Enabled by Electrical Pumping. Appl. Phys. Lett..

[142] Liang J., Yan Y., Zhao Y.S. (2021). Organic Microlaser Arrays: From Materials Engineering to Optoelectronic Applications. Acc. Mater. Res..

[143] Guo X., Zhen S., Ouyang T., Zhou S., Pan Q., Yang D., Chen J., Dong G., Zhao Z., Qiu J. (2021). An Organic Microlaser Based on an Aggregation-Induced Emission Fluorophore for Tensile Strain Sensing. J. Mater. Chem. C.

[144] Ta V.D., Nguyen T.V., Pham Q.V., Nguyen T.V. (2020). Biocompatible Microlasers Based on Polyvinyl Alcohol Microspheres. Opt. Commun..

[145] Zhao S., Li G., Peng X., Ma J., Yin Z., Zhao Q. (2022). Ultralow-Threshold Green Fluorescent Protein Laser Based on High Q Microbubble Resonators. Opt. Express.

[146] Ma J., Zhao S., Peng X., Li G., Wang Y., Zhang B., Zhao Q. (2023). An mCherry Biolaser Based on Microbubble Cavity with Ultra-Low Threshold. Appl. Phys. Lett..

[147] Hirano M., Ando R., Shimozono S., Sugiyama M., Takeda N., Kurokawa H., Deguchi R., Endo K., Haga K., Takai-Todaka R. (2022). A Highly Photostable and Bright Green Fluorescent Protein. Nat. Biotechnol..

[148] Zhang Y., Yang Y., Ding S., Zeng X., Li T., Hu Y., Lu S. (2025). Exploring Carbon Dots for Biological Lasers. Adv. Mater..

[149] Protyai M.I.H., Bin Rashid A. (2024). A Comprehensive Overview of Recent Progress in MXene-Based Polymer Composites: Their Fabrication Processes, Advanced Applications, and Prospects. Heliyon.

[150] Lau K.Y., Liu X., Qiu J. (2022). MXene Saturable Absorbers in Mode-Locked Fiber Laser. Laser Photonics Rev..

[151] Lee K., Choi J., Kwon S., Kim J., Woo T., Ryu J., Jung J., Lee J.H. (2023). Passively Mode-Locked Erbium-Doped Fiber Laser by a Mo2TiC2 MXene Saturable Absorber. Optik.

[152] Cai L., Liu Y., Jing H., Li L., Gao K., Zhang J., Wang Y., Huang S. (2026). MXene Functionalized Whispering Gallery Mode Microbubble Resonator for Pb2+ Sensing. Microchem. J..

[153] Sharbirin A.S., Roy S., Tran T.T., Akhtar S., Singh J., Duong D.L., Kim J. (2022). Light-Emitting Ti_2_ N (MXene) Quantum Dots: Synthesis, Characterization and Theoretical Calculations. J. Mater. Chem. C.

[154] Sharbirin A.S., Akhtar S., Kim J. (2021). Light-Emitting MXene Quantum Dots. OEA.

[155] Lin C., Song X., Ye W., Liu T., Rong M., Niu L. (2024). Recent Progress in Optical Sensors Based on MXenes Quantum Dots and MXenes Nanosheets. J. Anal. Test..

[156] Sariga, Babu A.M., Kumar S., Rajeev R., Thadathil D.A., Varghese A. (2023). New Horizons in the Synthesis, Properties, and Applications of MXene Quantum Dots. Adv. Mater. Interfaces.

[157] Zhao G., Lin J., Fu B., Gao R., Li C., Yao N., Guan J., Li M., Wang M., Qiao L. (2024). Integrated Multi-Color Raman Microlasers with Ultra-Low Pump Levels in Single High-Q Lithium Niobate Microdisks. Laser Photonics Rev..

[158] Cho S., Martino N., Yun S.-H. (2025). Half-Wave Nanolasers and Intracellular Plasmonic Lasing Particles. Nat. Nanotechnol..

[159] Saito Y., Oikawa M., Nakazawa H., Niide T., Kameda T., Tsuda K., Umetsu M. (2018). Machine-Learning-Guided Mutagenesis for Directed Evolution of Fluorescent Proteins. ACS Synth. Biol..

[160] Notin P., Rollins N., Gal Y., Sander C., Marks D. (2024). Machine Learning for Functional Protein Design. Nat. Biotechnol..

[161] Zhu Y., Fang J., Ahmed S.A.H., Zhang T., Zeng S., Liao J.-Y., Ma Z., Qian L. (2025). A Modular Artificial Intelligence Framework to Facilitate Fluorophore Design. Nat. Commun..

[162] Shahzad K., Mardare A.I., Hassel A.W. (2024). Accelerating Materials Discovery: Combinatorial Synthesis, High-Throughput Characterization, and Computational Advances. Sci. Technol. Adv. Mater. Methods.

[163] Stroyuk O., Raievska O., Zahn D.R.T., Brabec C.J. (2025). Accelerated Discovery of Multinary Chalcogenide Quantum Dots: Combining Aqueous Chemistry with High-Throughput Experimentation and Machine Learning. Nano Futures.

[164] Kim G., Barbay S., Calvet L. (2025). Complex Image Classification with Micro Laser Neurons Integrated with DNN-Assisted Genetic Algorithm. Proceedings of the 2025 Conference on Lasers and Electro-Optics Europe & European Quantum Electronics Conference (CLEO/Europe-EQEC), Munich, Germany, 23 June 2025.

[165] Tom G., Schmid S.P., Baird S.G., Cao Y., Darvish K., Hao H., Lo S., Pablo-García S., Rajaonson E.M., Skreta M. (2024). Self-Driving Laboratories for Chemistry and Materials Science. Chem. Rev..

